# Reduced expression of SMAD4 in gliomas correlates with progression and survival of patients

**DOI:** 10.1186/1756-9966-30-70

**Published:** 2011-07-27

**Authors:** Shi-ming He, Zhen-wei Zhao, Yuan Wang, Ji-pei Zhao, Liang Wang, Fang Hou, Guo-dong Gao

**Affiliations:** 1 Department of Neurosurgery, Institute for functional neurosurgery P.L.A, TangDu Hospital, Fourth Military Medical University, Xi'an, 710038, PR China

**Keywords:** glioma, SMAD4, Immunochemistry assay, Quantitative real-time PCR, Western blot analysis, prognosis

## Abstract

**Background:**

To examine the expression of SMAD4 at gene and protein levels in glioma samples with different WHO grades and its association with survival.

**Methods:**

Two hundreds fifty-two glioma specimens and 42 normal control tissues were collected. Immunochemistry assay, quantitative real-time PCR and Western blot analysis were carried out to investigate the expression of SMAD4. Kaplan-Meier method and Cox's proportional hazards model were used in survival analysis.

**Results:**

Immunohistochemistry showed that SMAD4 expression was decreased in glioma. SMAD4 mRNA and protein levels were both lower in glioma compared to control on real-time PCR and Western blot analysis (both P < 0.001). In addition, its expression levels decrease from grade I to grade IV glioma according to the results of real-time PCR, immunohistochemistry analysis and Western blot. Moreover, the survival rate of SMAD4-positive patients was higher than that of SMAD4-negative patients. We further confirmed that the loss of SMAD4 was a significant and independent prognostic indicator in glioma by multivariate analysis.

**Conclusions:**

Our data provides convincing evidence for the first time that the reduced expression of SMAD4 at gene and protein levels is correlated with poor outcome in patients with glioma. SMAD4 may play an inhibitive role during the development of glioma and may be a potential prognosis predictor of glioma.

## 1. Introduction

Human gliomas are the most common primary intracranial tumors in adults. A grading scheme proposed by the WHO distinguishes four different grades of gliomas, of which glioblastoma multiforme (GBM) WHO grade IV is the most malignant variant with a median survival time of 1 year [1]. Many aggressive treatment approaches, such as postoperative radiation therapy and chemotherapy, have been used clinically. However, these approaches do not benefit all patients equally. Adverse effects of these approaches even dramatically deteriorate the quality-of-life of some patients. Therefore, individualized therapy should be considered as a valuable approach for patients with high-grade gliomas. Molecular profiling of gliomas may define the critical genetic alterations that underlie glioma pathogenesis and their marked resistance to therapy [2]. So elucidation of these critical molecular events will improve therapy and individualize therapeutic interventions for patients with gliomas.

Mothers against decapentaplegic homologue 4 (SMAD4), expressed ubiquitously in different human organ systems, was initially isolated as a tumor suppressor gene on chromosome 18q21.1 in pancreatic ductal adenocarcinomas [3]. The SMAD4 protein is the downstream mediator of transforming growth factor beta (TGF-β), which is an important multifunctional cytokine that regulates cell proliferation, differentiation and extracellular matrix production [4]. Conflicting data exist about the influence of SMAD4 on the development and progression of various human tumors. Papageorgis et al. reported that SMAD4 inactivation promotes malignancy and drug resistance of colon cancer [5]. The study of Sakellariou et al. found that SMAD4 may behave as a tumor promoter in low grade gastric cancer and the survival rates were significantly higher for patients with reduced SMAD4 expression, in cases of well- or moderately differentiated tumors [6]. In pancreatic cancer, inactivation of the SMAD4 gene through mutation occurs frequently in association with malignant progression [7]. In non-small-cell lung carcinoma, immunohistochemistry revealed that SMAD4 was expressed at high level in normal broncho-tracheal epithelium, but at low level in tumor tissues, and closely correlated with tumor lymph node metastasis [8]. Lv et al. also demonstrated that the hypo-expression level of SMAD4 was associated with the pathological stage, and lymph node metastasis of the patients with esophageal squamous cell carcinoma, however, it might not be the independent prognostic factor [9]. On the other hand, Sheehan et al. indicated that SMAD4 protein expression persists in prostatic adenocarcinomas compared with benign glands, with both nuclear and cytoplasmic overexpression correlating with prognostic variables indicative of aggressive tumor behavior [10]. Hiwatashi et al. also concluded that strong SMAD4 expression in hepatocellular carcinoma is likely to suggest poor prognosis of patients [11]. However, little is known about the expression level of SMAD4 or its prognostic significance in human gliomas.

In order to gain further insight into the status of SMAD4 in the progression of glioma, we used immunochemistry assay, quantitative real-time PCR and Western blot analysis to investigate the expression pattern of SMAD4 in glioma specimens and normal control brain tissues. Next, we analyzed the relationship between SMAD4 expression and the glioma stage as well as the survival of patients.

## 2. Materials and methods

### 2.1 Patients and Tissue Samples

This study was approved by the Research Ethics Committee of the Institute for functional neurosurgery P.L.A, TangDu Hospital, Fourth Military Medical University, Xi'an, P.R. China. Written informed consent was obtained from all of the patients. All specimens were handled and made anonymous according to the ethical and legal standards.

Fresh glioma specimens were obtained from 252 patients who underwent surgery between May 2002 and April 2005. None of the patients had received radiotherapy or chemotherapy prior to surgery. About 42 normal brain tissue samples were taken from patients who underwent surgery for reasons other than malignancy such as cerebral trauma. This served as the control. Tumors were histopathologically classified according to the WHO classification. Patient data included age, sex, date and type of initial operation, and details of the follow-up. Clinical information was obtained by reviewing the medical records on radiographic images, by telephone or written correspondence, and by review of death certificate. A patient was considered to have recurrent disease if this was revealed either by magnetic resonance imaging or the occurrence of new neurologic symptoms. Parts of the specimens were fixed in 10% formaldehyde and imbedded in paraffin for histological sections. Other parts were put into liquid N2 for 10 min, then into a -70°C ultra-freezer for mRNA and protein isolation. In the follow-up period, overall survival was measured from diagnosis to death or last follow-up.

### 2.2 Immunohistochemistry assay

Immunohistochemical assay was performed using the conventional immunoperoxidase technique according to the protocol of the Department of Neurosurgery, Institute for functional neurosurgery P.L.A, TangDu Hospital, Fourth Military Medical University, Xi'an, P.R. China. Briefly, following peroxidase blocking with 0.3% H2O2/methanol for 30 min, specimens were blocked with phosphate-buffered saline (PBS) containing 5% normal horse serum (Vector Laboratories Inc., Burlingame, CA, USA). All incubations with anti-SMAD4 antibody (clone B-8, Santa Cruz Biotechnology Inc, Heidelberg, Germany) at 1:50 dilution were carried out overnight at 4°C. Then the specimens were briefly washed in PBS and incubated at room temperature with the anti-mouse antibody and avidin-biotin peroxidase (Vector Laboratories Inc., Burlingame, CA, USA). The specimens were then washed in PBS and color-developed by diaminobenzidine solution (Dako Corporation, Carpinteria, CA, USA). After washing with water, specimens were counterstained with Meyer's hematoxylin (Sigma Chemical Co., St Louis, MO, USA). Normal brain tissues were used as control tissues and non-immune IgG was also used as negative control antibody for immunohistochemical staining.

Stained sections were observed under a microscope. Immunostaining was scored by two independent experienced pathologists, who were blinded to the clinicopathologic parameters and clinical outcomes of the patients. An immunoreactivity score system was applied as described previously [12]. The extensional standard was: (1) the number of positively stained cells <5% scored 0; 6-25% scored 1; 26-50% scored 2; 51-75% scored 3; >75% scored 4; (2) intensity of stain: colorless scored 0; pallide-flavens scored 1; yellow scored 2; brown scored 3. Multiply (1) and (2). The staining score was stratified as - (0 score, absent), + (1-4 score, weak), ++ (5-8 score, moderate) and +++ (9-12 score, strong) according to the proportion and intensity of positively stained cancer cells. Specimens were rescored if difference of scores from two pathologists was >3.

### 2.3 Quantitative real-time PCR

Total RNA purified from all 252 glioma tissues and 42 control brain tissues was prepared and reverse transcribed. Real-time monitoring of polymerase chain reactions (PCRs) was performed using the ABI 7900HT (Idaho Technology, Idaho Falls, ID, USA) and the SYBR green I dye (Biogene), which binds preferentially to double-stranded DNA. Fluorescence signals, which are proportional to the concentration of the PCR product, are measured at the end of each cycle and immediately displayed on a computer screen, permitting realtime monitoring of the PCR. The reaction is characterized by the point during cycling when amplification of PCR products is first detected, rather than the amount of PCR product accumulated after a fixed number of cycles. The higher the starting quantity of the template, the earlier a significant increase in fluorescence is observed. The threshold cycle is defined as the fractional cycle number at which fluorescence passes a fixed threshold above the baseline. The primers 5'- TAT TAA GCA TGC TAT ACA ATC TG -3' and 5'- CTT CCA CCC AGA TTT CAA TTC -3' were used to amplify 332-bp transcripts of SMAD4 and the primers 5'- GGT GGC TTT TAG GAT GGC AAG -3' and 5'- ACT GGA ACG GTG AAG GTG ACA G -3' were used to amplify 161-bp transcripts of β-actin. All primers were synthesized by Sangon Co. (Shanghai, China). The PCR profile consisted of an initial melting step of 1 min at 94°C, followed by 38 cycles of 15 s at 94°C, 15 s at 56°C and 45 s at 72°C, and a final elongation step of 10 min at 72°C.

Fluorescence data were converted into cycle threshold measurements using the SDS system software and exported to Microsoft Excel. SMAD4 mRNA levels were compared to β-actin. Thermal dissociation plots were examined for biphasic melting curves, indicative of whether primer-dimers or other nonspecific products could be contributing to the amplification signal.

### 2.4. Western blot analysis

Glioma and normal brain tissues were homogenized in lysis buffer [PBS, 1% nonidet P-40 (NP-40), 0.5% sodium deoxycholate, 0.1% sodium dodecyl sulfate (SDS), 100 ug/ml aprotinin, 100 μg/ml phenylmethylsulfonyl fluoride (PMSF), Sodium orthovanadate] at 4°C throughout all procedures, and sonicated for 70 s, then add 300 μg PMSF per gram of tissue and incubate on ice for 30 min, followed by centrifugation at 15,000 rpm for 20 min at 4°C. The protein content was determined according to Bradford's method (Bradford 1976), with bovine serum albumin used as a standard. Protein samples (30 μg) were boiled with 2 × sample buffer containing 5% β-mercaptoethanol for 5 min, separated by size on 15% polyacrylamide gel under SDS denaturing conditions, and transferred to a nitrocellucose membrane at 90 V for 2 h. The nitrocellulose membranes were stained with ponceau S to assess the efficiency of transfer. Non-specifi c binding was blocked by incubation in block buffer (5% non-fat dry milk, 0.05% Tween-20, 1 × tris-Cl-buffered saline) overnight at 4°C, The membranes were hybridized with mouse monoclonal antibody recognizing SMAD4 (sc-7966, Santa Cruz Biotechnology, Inc., Santa Cruz, CA), then incubated with a horseradish peroxidase-labeled goat anti-mouse IgG (1: 500). The bound secondary antibody was detected by enhanced chemiluminescence (Amersham Life Science, Little Chalfont, UK). Housekeeping protein β-actin was used as a loading control. Positive immunoreactive bands were quantified densitometrically (Leica Q500IW image analysis system) and expressed as ratio of SMAD4 to β-actin in optical density units.

### 2.5 Statistical analysis

All computations were carried out using the software of SPSS version13.0 for Windows (SPSS Inc, IL, USA). The rank sum test was used to analyze the ranked data. The measurement data were analyzed by one-way ANOVA. Randomized block design ANOVA was used to analyze the statistical difference among different tissue types. In the analysis of glioma morbidity for all patients, we used the Kaplan-Meier estimator and univariate Cox regression analysis to assess the marginal effect of each factor. The differences between groups were tested by log-rank analyses. The joint effect of different factors was assessed using multivariate Cox regression. A Spearman's analysis was carried out to analyze the correlation between SMAD4 mRNA and protein expression levels. Differences were considered statistically significant when *p *was less than 0.05.

## 3. Results

### 3.1 SMAD4 protein levels in glioma tissues by immunohistochemistry assay and survival analysis

**Figure 1:**
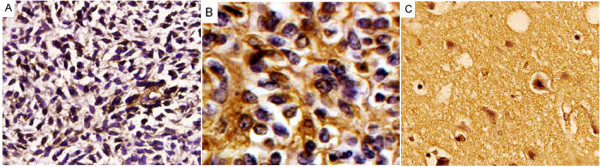
**Immunohistochemical staining of SMAD4 protein in tumor cells of GBM (A) and astrocytoma (B) (Original magnification ×400)**. Staining for this antigen is described in Materials and Methods. Positive staining of SMAD4 is seen in the cytoplasm and/or nuclei of tumors cells and is more abundant in the low- (B) than the high-grade (A) tumors. Intensively positive expression of SMAD4 (C) was observed in normal brain tissues.

SMAD4 expression was studied in a total of 252 glioma specimens of which 113 were low grade glioma (grade I and II) and 139 were high grade (grade III and IV). About 42 specimens taken from normal brain tissue served as control group. Based on immunohistochemistry analysis, positive staining for SMAD4 was mainly observed in the cytoplasm and to a lesser degree in the nuclei of cancer cells. The representative photographs were shown in Figure 1. Among the glioma specimens, 138 (54.8%) glioma specimens were positively stained, and 114 (45.2%) glioma specimens were negatively stained. Among the control specimens, 34 (81.0%) were positively stained, and 8 (19.0%) were negatively stained. We also found a significant decrease of SMAD4 expression in glioma compared with normal brain tissues (P < 0.001).

In addition, SMAD4 expression was not significantly affected by the gender and age (both P > 0.05) of the patients. In contrast, the SMAD4 expression was the closely correlated with WHO grade (Table 1; P = 0.008), as well as Karnofsky performance Status (KPS) (Table 1; P < 0.001).

**Table 1:** SMAD4 expression in human glioma tissues with different clinical-pathological features

Clinicopathological features	No. of cases	SMAD4 (n)				P
		-	+	++	+++	
**WHO grade**		114	60	51	27	
I	53	12	16	13	12	0.008
II	60	17	21	15	7	
III	62	34	12	11	5	
IV	77	51	11	12	3	
**Age**						
<55	152	65	39	31	17	NS
≥55	100	49	21	20	10	
**Gender**						
Male	138	57	36	30	15	NS
Female	114	57	24	21	12	
**KPS**						
<80	135	81	25	21	8	<0.001
≥80	117	33	35	30	19	

**Figure 2:**
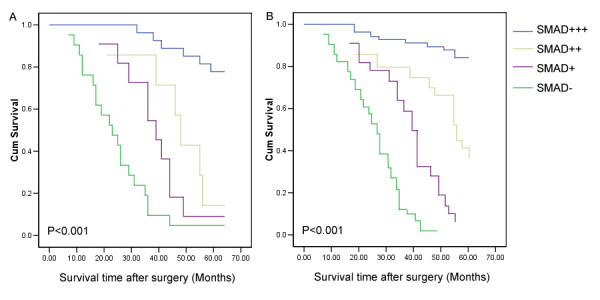
**Postoperative survival curves for patterns of patients with glioma and SMAD4 expression**. (A) Kaplan-Meier postoperative survival curve for patterns of patients with glioma and SMAD4 expression. Unadjusted RR of SMAD4-negative (-), weak positive (+), moderate positive (++) and strong positive (+++) groups were 1.0, 0.4, 0.08 and 0.02, respectively (P < 0.001). (B) Cox proportional hazards model after adjusting for age, gender and grade. SMAD4 might be an independent predictor of survival, without consideration of age, gender or grade. Adjusted RR of SMAD4-negative (-), weak positive (+), moderate positive (++) and strong positive (+++) groups were 1.0, 0.4, 0.2 and 0.04, respectively (P < 0.001).

Moreover, we reviewed clinical information of these SMAD4-positive or -negative glioma patients. During the follow-up period, 197 of the 252 glioma patients (78.2%) had died (108 from the SMAD4-negative group and 142 from the SMAD4-positive group). As determined by the log-rank test, the survival rate of patients without SMAD4 staining was lower than those showing SMAD4 positive staining (P < 0.001; Figure 2A). The median survival time of patients with strong positive (+++) expression of SMAD4 could not be estimated by statistical analysis because all patients survived better than the overall median level, and those patients with moderate positive (++), weak positive (+) and negative expression of SMAD4 were 22.8 ± 1.3 months, 13.2 ± 1.6 months and 8.0 ± 0.5 months (log-rank test: P < 0.001).

Furthermore, Figure 2B shows the post-operative survival curve of patients with glioma and SMAD4 expression after adjusting for age, gender, WHO grade and KPS. By multivariate analysis, the loss of SMAD4 expression was a significant and independent prognostic indicator for patients with glioma besides age, WHO grade and KPS. The Cox proportional hazards model showed that lower SMAD4 expression was associated with poor overall survival.

### 3.2 Quantitative analysis of SMAD4 protein expression based on WHO grade in gliomas

**Figure 3:**
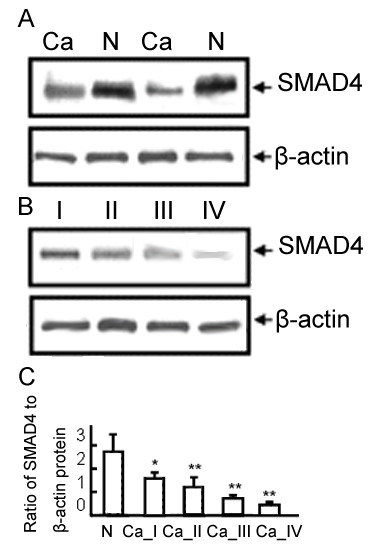
**Expression of SMAD4 protein in glioma and normal brain tissues by Western blot analysis**. (A) SMAD4 expression levels in glioma and normal brain tissues. (B) SMAD4 expression levels in glioma with different WHO grades. (C) SMAD4 expression levels in normal brain tissues and glioma with different WHO grades. 'N' refers to normal brain tissues; 'Ca' refers to glioma tissues; 'Ca_ I'~' Ca_ IV' refer to glioma tissues with WHO grade I~ IV. β-actin was used as a control for equal protein loading. Values are means ± SD. '*', p < 0.05, comparison with normal brain tissues; '**', p < 0.001, comparison with normal brain tissues.

As the results of Western blot analysis, we found that SMAD4 protein expression tended to increase from the glioma to the normal tissue (Figure 3A, C). We also investigated whether the expression of SMAD4 correlated with the WHO grade. SMAD4 expression was highest in grade I and lowest in grade IV (Figure 3B, C). This result agreed with the findings of the immunohistochemistry analysis and indicated a close correlation of SMAD4 protein expression with WHO grade.

### 3.3 Quantitative analysis of SMAD4 gene expression in glioma

We determined the mRNA expression of SMAD4 normalized to β-actin by real-time PCR. As shown in Table 2, there was a conspicuous decrease in the expression of SMAD4 mRNA from the control brain tissues to glioma tissues (P < 0.001). We further analyzed the expression of SMAD4 mRNA based on KPS and WHO grade. Interestingly, SMAD4 mRNA expression decreased in patients whose KPS lower than 80 (P < 0.001) and also decreased with advancement of WHO grade I to grade IV (P < 0.01). There was a significant positive correlation between the expression of SMAD4 mRNA and protein expression levels from the same glioma tissues (rs = 0.886, P < 0.001).

**Table 2:** Statistics of SMAD4 mRNA levels in glioma

	No. of cases	SMAD mean (SD)	P
**Tissue type**			
Control	42	2.096 (0.338)	<0.01
Glioma	252	0.861 (0.223)	
**WHO grade**			
I	53	1.517 (0.097)	<0.001
II	60	1.205 (0.136)	
III	62	0.615 (0.412)	
IV	77	0.339 (0.036)	
**KPS**			
<80	135	0.372 (0.113)	<0.001
≥80	117	1.425 (0.375)	

## 4. Discussion

In the current study, we investigated the expression of SMAD4 in 252 cases of human glioma and compared the expression with tumor grade and survival rates of patients. Our data demonstrated that SMAD4 protein was decreased in glioma compared to normal brain tissue. SMAD4 mRNA expression was also reduced in glioma compared with control normal brain tissue. We found a decreased trend of both SMAD4 protein level and mRNA level from WHO grade I to WHO grade IV glioma. These results suggest that the transcriptional repression of human SMAD4 might participate in the carcinogenesis and progression of glioma. SMAD4 may have an important role during the genesis or progression of glioma.

SMAD proteins are the key intracellular mediators of transcriptional responses to TGF-β signaling which is altered in various tumors [13]. They consistently transmit the TGF-β signal from the cell membrane to the nucleus. The mammalian SMAD family consists of eight members, which can be divided into three groups according to their function: receptor-activated SMADs, commonmediated SMADs, and inhibitory SMADs [14]. SMAD4 is one of the commonmediated SMADs and, in general, SMAD4 is a central component of the TGF-β/SMAD pathway and is expressed in different human organ systems. TGF-β binds to homodimers of the TGF-β type II receptor (TβRII) which recruits and activates homodimers of TGF-β type I receptor (TβRI) serine/threonine kinase. Activated TβRI phosphorylates SMAD2 or SMAD3 which heterodimerize with SMAD4. These heterocomplexes translocate into the nucleus where they bind DNA and regulate TGF-β dependent gene expression [15]. Deletion or degradation of SMAD4 in tumors could specifically inhibit the tumor suppressor effect of TGF-β. SMAD4 alteration has been associated with specific loss of TGF-β induced growth resulting in increased angiogenesis and loss of epithelial integrity [16]. Recent studies have shown that SMAD4 inactivation is associated with the advanced disease state of various human tumors, including pancreatic carcinoma, esophageal carcinoma, colorectal carcinoma, renal cell carcinoma, as well as breast carcinoma [17-20]. Our results confirm that SMAD4 is downregulated during tumor progression. Kjellman et al. [21] analyzed the mRNA expression of TGF-β1, TGF-β2, TGF-β3, the TGF-β receptors type I (TβR-I) and type II (TβR-II), SMAD2, SMAD3, and SMAD4. Their data suggested that TGF-β normally up-regulates the TGF-β receptors, and TβR-I and TβR-II showed stronger expression in all gliomas when compared to normal tissues. However, the mRNA expression of SMAD2, SMAD3, and SMAD4 was decreased in GBM, which was consistent with the results of our study.

We further analyzed the correlation of SMAD4 expression and survival rates of patients. Our data indicated that nearly 55% of glioma cases showed positive staining for SMAD4. The survival rate of patients without SMAD4 staining was lower than those showing SMAD4-positive staining. Kaplan-Meier analysis of the survival curves showed a significantly worse overall survival for patients whose tumors had low SMAD4 levels, indicating that low SMAD4 protein level is a marker of poor prognosis for patients with glioma. Moreover, multivariate analysis showed low SMAD4 expression to be a marker of worse outcome independent of the known clinical prognostic indicators such as age, KPS and grade. These data suggest that low expression of SMAD4 is correlated with a worse outcome of patients with glioma. Thus, SMAD4 might be an independent predictor of survival for glioma patients. In our study, which consisted of a large sample (n = 252), SMAD4 expression was analyzed by immunohistochemistry, real-time PCR and Western blot analysis. Thus, a large sample size, a good methodology and a detailed clinical follow-up in our study make it reliable.

In conclusion, our data provides convincing evidence for the first time that the reduced expression of SMAD4 at gene and protein levels is correlated with poor outcome in patients with glioma. SMAD4 may play an inhibitive role during the development of glioma and may be a potential prognosis predictor of glioma.

## Competing interests

The authors declare that they have no competing interests.

## Authors' contributions

S-MH and Z-WZ carried out the Immunochemistry assay and Quantitative real-time PCR. SMH also drafted the manuscript. YW carried out the Western blot analysis and drafted the manuscript. J-PZ, LW and FH participated in the survival analysis. G-DG conceived of the study, and participated in its design and coordination. All authors read and approved the final manuscript.
